# Proliferation and odontogenic differentiation of *BMP2* gene-transfected stem cells from human tooth apical papilla: An *in vitro* study

**DOI:** 10.3892/ijmm.2014.1862

**Published:** 2014-07-24

**Authors:** WEN ZHANG, XIAOLEI ZHANG, JUNQI LING, WEI LIU, XINCHUN ZHANG, JINGLEI MA, JIANMAO ZHENG

**Affiliations:** Department of Operative Dentistry and Endodontics, Guanghua School and Hospital of Stomatology and Guangdong Province Key Laboratory of Stomatology, Sun Yat-sen University, Guangzhou 510080, P.R. China

**Keywords:** stem cells from apical papilla, bone morphogenetic protein 2, gene transfection, proliferation, odontogenic differentiation

## Abstract

Stem cells from the apical papilla (SCAP) have odontogenic potential, which plays a pivotal role in the root dentin development of permanent teeth. Human bone morphogenetic protein 2 (*BMP2*) is a well-known gene that participates in regulating the odontogenic differentiation of dental tissue-derived stem cells. However, little is known regarding the effects of the *BMP2* gene on the proliferation and odontogenic differentiation of SCAP. This study aimed to evaluate the odontogenic differentiation potential of lentiviral-mediated *BMP2* gene-transfected human SCAP (SCAP/BMP2) *in vitro*. SCAP were isolated by enzymatic dissociation of human teeth apical papillae. The multipotential of SCAP was verified by their osteogenic and adipogenic differentiation characteristics. The phenotype of SCAP was evaluated by flow cytometry (FCM). The proliferation status of the blank vector-transfected SCAP (SCAP/Vector) and SCAP/BMP2 was analyzed by a cell counting kit-8 (CCK-8). Odontogenic genes, including alkaline phosphatase (ALP), osteocalcin (OCN), dentin sialophosphoprotein (DSPP) and dentin matrix protein 1 (DMP1) of the two groups of cells were evaluated by quantitative polymerase chain reaction (qPCR). ALP staining and alizarin red (AR) staining of the cells was performed on the 16th day after transfection. *In vitro* results of CCK-8, qPCR, ALP and AR staining demonstrated that: i) SCAP/BMP2 had a comparable proliferation rate to SCAP/Vector; ii) SCAP/BMP2 presented significantly better potential to differentiate into odontoblasts compared to SCAP/Vector by upregulating *ALP*, *OCN*, *DSPP* and *DMP1* genes; iii) more ALP granules and mineralized deposits were formed by SCAP/BMP2 as compared to SCAP/Vector. The results suggested that lentiviral-mediated *BMP2* gene transfection enhances the odontogenic differentiation capacity of human SCAP *in vitro*.

## Introduction

In 2006, Sonoyama *et al* isolated a new population of mesenchymal stem cells from the root apical papilla of human tooth, which were designated as stem cells from apical papilla (SCAP) (1). SCAP expressed mesenchymal stem cell phenotypes, including STRO-1 and CD146, and a specific surface marker, i.e., CD24 (2–5). SCAP are considered the source of odontoblasts, which are responsible for the development of root dentin (3–7). In a minipig model, tooth root development was ceased when the apical papilla was surgically removed, whereas root development was completed when the apical papilla was conserved (6). SCAP have a significantly higher proliferation rate and mineralization capacity than dental pulp stem cells (DPSCs) (3,7). Findings of previous studies demonstrated that the proliferation ratio of SCAP was approximately 2 times greater than that of DPSCs *in vitro* (3,5). Additionally, both DPSCs and SCAP presented an initiative potential for mineralization and osteo/odontogenic gene expression, including alkaline phosphatase (ALP), osteocalcin (OCN), bone sialoprotein (BSP) and dentin sialophosphoprotein (DSPP) (3). Furthermore, the mineralization ratio of SCAP was significantly higher than that of DPSCs with osteo/odontogenic stimuli, which resulted in increased amounts of mineralized deposits in SCAP (3,5,7). As a novel seed cell for dental regeneration, SCAP are believed to have broad prospects. However, the genes that regulate the odontogenic differentiation of SCAP remain unclear.

Bone morphogenetic protein 2 (*BMP2*) is a key gene for modulating odontogenic differentiation in tooth development (8–10). It is involved in the regulation of odontogenic differentiation of dental pulp cells, and in the control of the mineralization processes of the dentin matrix (11). Co-cultured with recombinant human BMP2 (rhBMP2) medium, the odontogenic differentiation of DPSCs was promoted, resulting in an increased gene expression of DSPP *in vitro*, and an enhanced reparative dentin formation on amputated pulp (12). Yang *et al* constructed human *BMP2* gene-transfected DPSCs (DPSCs/BMP2), and reported that the osteo/odontogenic differentiation genes, including ALP, OCN, collagen type I (Col I), BSP, DSPP and dentin matrix protein 1 (DMP1), were significantly enhanced at different time points, compared with DPSCs (13–15). Furthermore, rhBMP2-treated DPSCs presented significantly reduced proliferation compared with the control (16). However, the effects of BMP2 on the odontogenic differentiation of SCAP remain to be elucidated.

In this study, *BMP2* gene was transfected into SCAP by lentiviral-mediated transfection to construct SCAP/BMP2. The proliferation capacity of SCAP/BMP2 was analyzed by CCK-8. The odontogenic differentiation potential of SCAP/BMP2 was evaluated by quantitative polymerase chain reaction (qPCR), ALP staining and alizarin red (AR) staining. We hypothesized that SCAP/BMP2 have greater odontogenic differentiation potential as compared to SCAP/Vector, when cultured without osteo/odontogenic stimuli. The results of the present study suggested the effects of the *BMP2* gene on the odontogenic differentiation of SCAP.

## Materials and methods

### Isolation and culture of SCAP

The experiments were performed with the approval of the Ethics Committee of Guanghua School and Hospital of Stomatology, Sun Yat-sen University (Guangzhou, China). Human impacted wisdom teeth with immature roots were collected from 16- to 18-year-old patients at the Department of Oral Maxillofacial Surgery of the Guanghua Hospital of Stomatology. The extracted third molars were carefully rinsed by phosphate-buffered saline (PBS) with 100 U/ml penicillin and 100 mg/ml streptomycin, and then temporarily maintained in Hanks’ solution. The root apical papillae were gently separated from teeth roots, minced and digested by 3 mg/ml type I collagenase (Worthington Biochemical Co., Freehold, NJ, USA) and 4 mg/ml dispase (Gibco Life Technologies, Beijing, China) for 1 h at 37°C. Single-cell suspension was obtained by passing through a 70 μm strainer (BD Biosciences, Bedford, MA, USA). These isolated SCAP with 5×10^4^/well density were seeded in 6-well plates (Corning Life Sciences, Oneonta, NY, USA) containing α-MEM (Invitrogen, Hong Kong, China) supplemented with 10% FBS (Gibco Life Technologies Australia Pty Ltd., Mulgrave Victoria, Australia), 100 U/ml penicillin-G and 100 mg/ml streptomycin, and then cultured with 5% CO_2_ at 37°C.

### Differentiation stimulation

The multipotential of SCAP was confirmed by osteogenic and adipogenic differentiation induction. Briefly, SCAP were cultured with OriCell™ Human Mesenchymal Stem Cell Osteogenic Differentiation Medium and OriCell Human Mesenchymal Stem Cell Adipogenic Differentiation Medium (both from Cyagen Biosciences Inc., Guangzhou, China) for 32 and 8 days, respectively. AR solution and Oil Red O reagent kit (Jiancheng Bioengineering Institute, Nanjing, China) were used to visualize the mineralized nodules and lipid droplets, respectively.

### Flow cytometry (FCM) analysis

The phenotypes of the passage 2 SCAP were evaluated by FCM for the expression of STRO-1/Alexa Fluor 647-APC (BioLegend, San Diego, CA, USA), CD146/PE, CD24/FITC and CD45/FITC (all from BD Pharmingen, San Diego, CA, USA).

### Construction of recombinant lentivirus plasmid

Human *BMP2* gene primers were designed by Oligo 7.0 software (Molecular Biology Insights, Inc., Plymouth, MN, USA) according to the NCBI GenBank no. KC294426.1. PCR primers were designed as follows: forward, GCCGAATTCA TGGTGGCCGGGACCCGCTG (the underlined is tje *Eco*RI site) and reverse, GCCGGATCCCTAGCGACACCCACAAC CCTC (the underlined is the *Bam*HI site). For PCR amplification, the primers were processed with a pre-denaturation step (95°C for 3 min), followed by 30 cycles of denaturation (95°C for 15 sec), annealing (55°C for 30 sec), and extension (72°C for 1 min), then stored at 72°C for 7 min prior to termination of the amplification action at 4°C for 10 min. The amplified primers were subsequently treated with *Eco*RI and *Bam*HI, and the fragment containing human *BMP2* gene was combined into the lentivirus vector pCDH-CMV-MCS-EF1-copGFP (pCDH; System Biosciences, Mountain View, CA, USA) to construct the recombinant plasmid, i.e., pCDH-BMP2.

### Lentiviral-mediated gene transfection of SCAP

A volume of 5 ml 293FT cell (System Biosciences) suspensions with 2.4×10^5^ cells/ml density was bred on a 10 cm dish 2 days prior to transfection, and was cultured by DMEM (Invitrogen) with 10% FBS. The recombinant plasmid pCDH-BMP2, packaging plasmid psPAX.2 and envelope plasmid pMD2.G (the latter two from Cyagen) were co-transfected into 293FT cells by Lipofectamine 2000 (Invitrogen, Carlsbad, CA, USA) (17,18). The supernatant of 293FT cells was assembled after 48 h, centrifuged at 1,000 × g 37°C for 10 min, and filtered by 0.2 μm syringe filter (Millipore Corp., Bedford, MA, USA). To obtain an optimum multiplicity of infection (MOI), SCAP were infected with pCDH-BMP2 plasmid at different MOI values (5, 10, 20, 50 and 70) to obtain SCAP/BMP2. Green fluorescent protein (GFP) fluoresce of SCAP/BMP2 was assessed under a fluorescence microscope (Carl Zeiss Microimaging GmbH, Gottingen, Germany), and the transfection efficiency was evaluated by the GFP expression proportion of the cells. SCAP/Vector was constructed by infecting SCAP with blank vector and served as the control. The *BMP2* gene expression of SCAP/Vector and SCAP/BMP2 was assessed by qPCR and western blot analysis 4 days after transfection. SCAP/Vector and SCAP/BMP2 were bred in 25 cm^2^ culture flasks containing α-MEM with 10% FBS, and cultured with 5% CO_2_ at 37°C. The culture medium was changed at 48-h intervals.

### Cell proliferation

SCAP/Vector and SCAP/BMP2 were seeded in 96-well plates with a density of 2×10^3^ cells/well and were cultured in α-MEM with 10% FBS. The cell proliferation rate was analyzed using a cell counting kit-8 (CCK-8; Dojindo, Tokyo, Japan) on the 1st, 2nd, 4th and 8th day following gene transfection.

### qPCR analysis

The total RNA of the SCAP/Vector and SCAP/BMP2 was extracted using TRIzol (Invitrogen) on the 1st, 4th, 8th, and 16th day following gene transfection. The RNA quantity was assessed by a spectrophotometer (Model 400-MR; Varian Inc., Palo Alto, CA, USA). For each sample, 2 μg RNA was utilized to synthesize cDNA by RevertAid First Strand cDNA Synthesis kit (Thermo Fisher Scientific Inc., Beijing, China). Real-time reaction was performed by iQ SYBR-Green Supermix and regulated by spectrofluorimetric thermal iCycler iQ5 (both from Bio-Rad, Hercules, CA, USA). The BMP2 and odontogenic differentiation genes, including *ALP*, *OCN*, *DSPP* and *DMP1*, were assessed by qPCR. Each sample was assessed 3 times. Glyceraldehyde-3-phosphate dehydrogenase (*GAPDH*) was selected as the housekeeping gene (Table I). The gene-specific primers were amplified with a denaturation step (95°C for 3 min); followed by 39 cycles of denaturation (95°C for 10 sec), annealing (55°C for 10 sec), and extension (72°C for 30 sec).

### Western blot analysis

Western blot analysis was performed as described in a previous study (19). Briefly, the total proteins were calculated by a Bio-Rad Blue protein assay (Bio-Rad Laboratories, Richmond, CA, USA). Protein was distinguished with rabbit polyclonal antibody against human BMP2 (1:500 dilution; Abcam, Cambridge, UK) and human GAPDH (1:5,000 dilution).

### ALP and AR staining

SCAP/Vector and SCAP/BMP2 were cultured in 6-well plates at the initial density of 5×10^4^ cells/well, in α-MEM containing 10% FBS. After 16 days incubation, an ALP staining kit (Jiancheng Bioengineering Institute) was applied to demarcate ALP granules. Microscopic images were captured at a magnification of ×100, ×200, ×400 and ×800, respectively. The AR staining solution was used to demarcate mineralized deposits. AR staining images were captured at a magnification of ×0 and ×200.

### Statistical analysis

Data of cell proliferation and odontogenic differentiation gene expression were expressed as the mean ± standard deviation. A two-way ANOVA test was used to analyze the differences between SCAP/Vector and SCAP/BMP2 (SPSS,Inc., Chicago, IL, USA). The significance was set as P<0.05.

## Results

### Multipotential and phenotypic characteristics of SCAP

**Figure 1:**
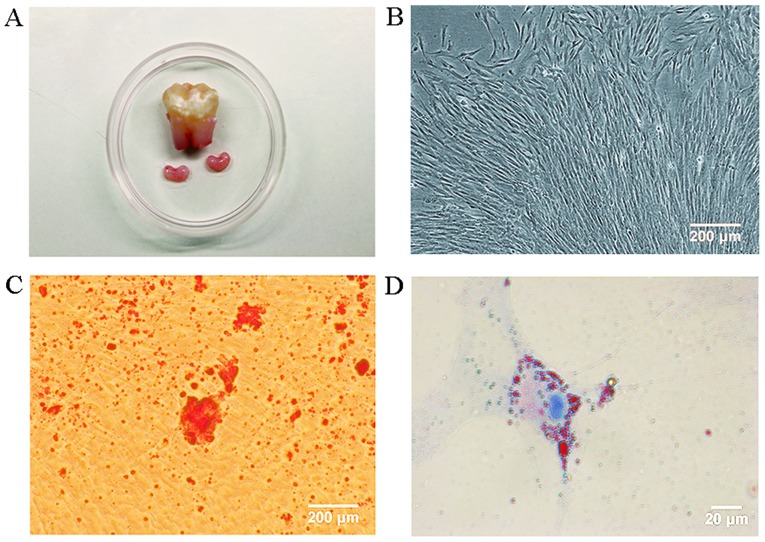
Isolation and multilineage differentiation of stem cells from apical papilla (SCAP). (A) Apical papillae that were isolated from the 3rd molars were kidney-shaped. (B) SCAP showed typical spindle-shaped morphology. (C) Mineralized deposits that formed by SCAP after 32 days osteogenic induction differentiation showed positive alizarin red staining (AR). (D) Lipid droplets that formed by SCAP after 8 days adipogenic induction differentiation showed positive Oil Red O staining.

**Figure 2:**
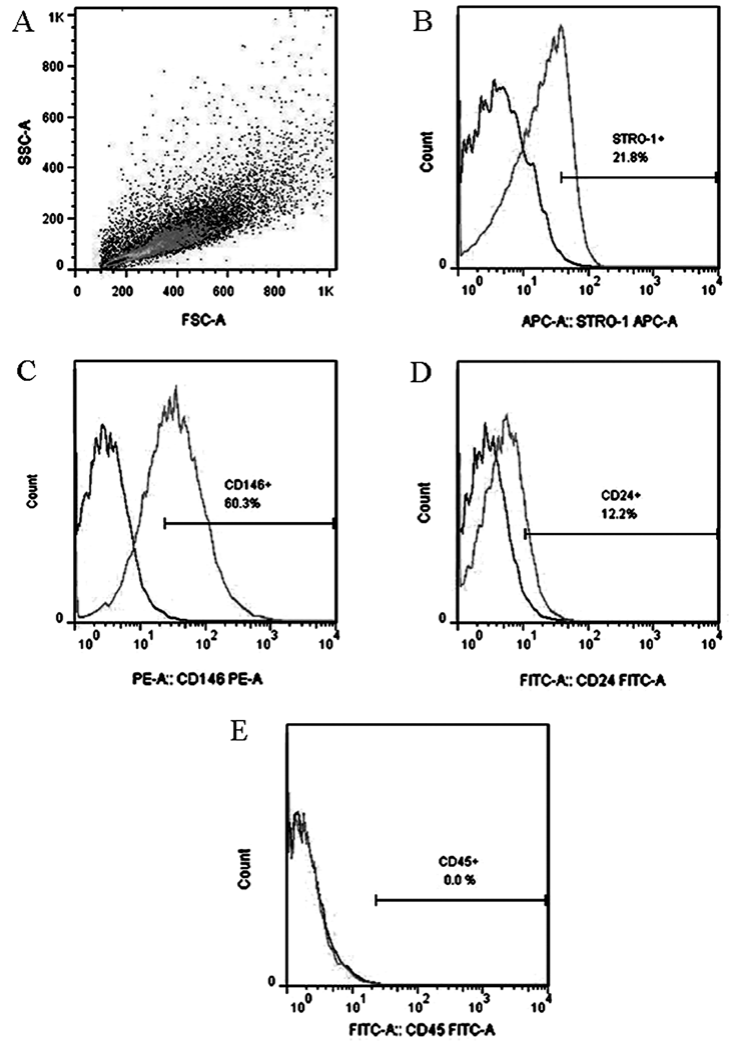
Flow cytometry (FCM) of stem cells from apical papilla (SCAP) Representative FCM results of SCAP are shown. (A) Forward vs. side scatter diagrams (FSC/SSC) of SCAP. (B–D) SCAP had a strong positive expression of STRO-1, CD146 and CD24. (E) SCAP had a negative expression of CD45.

The apical papillae were kidney-shaped (Fig. 1A). Isolated cells from apical papilla presented a spindle appearance with extending cytoplasmic processes (Fig. 1B). After 32 days osteogenic induction of SCAP, a mass of mineralized deposits formed and presented positive Alizarin Red staining (Fig. 1C). After 8 days adipogenic induction, a few lipid droplets formed and showed positive Oil Red O staining (Fig. 1D). The representative FCM results confirmed that the freshly isolated SCAP had a typical FSC/SSC character (Fig. 2A) as reported previously (3). The isolated cells expressed mesenchymal stem cell (MSC) phenotypes, including STRO-1 and CD146, and a specific phenotype CD24 (Fig. 2B–D). The expression of CD45, a hematopoietic stem cell character, was negative (Fig. 2E).

### Transfection efficiency

**Figure 3:**
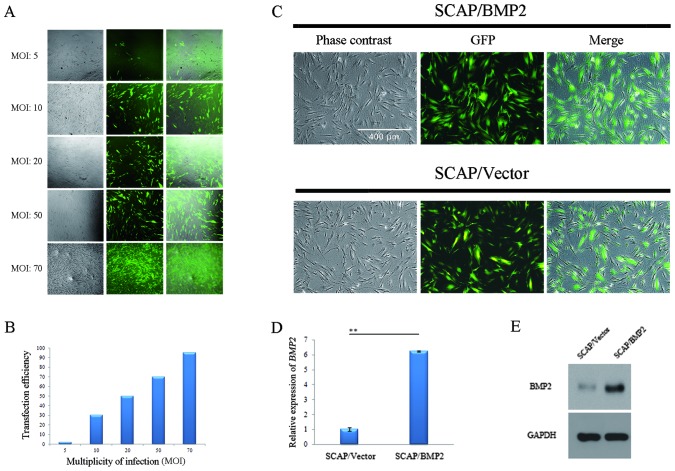
Transfection efficiency. (A–B) Forty-eight hours after transfection, the green fluorescent protein (GFP) expression improved with the increased optimum multiplicity of infection (MOI), and the transfection efficiency achieved >90% at a MOI of 70. (C) The GFP fluorescence was observed in >90% stem cells from apical papilla (SCAP)/Vector and SCAP/bone morphogenetic protein 2 (BMP2) 4 days after transfection. (D) The relative expression of the *BMP2* gene between SCAP/Vector and SCAP/BMP2 was tested by quantitative polymerase chain reaction (qPCR) 4 days after transfection. The *BMP2* gene relative expression was statistically higher in SCAP/BMP2 compared with SCAP/Vector, and the significance was marked as ^**^P<0.01. (E) The BMP2 expression of SCAP/Vector and SCAP/BMP2 was assessed by western blot analysis 4 days after transfection. The BMP2 expression was significantly upregulated in SCAP/BMP2 compared with SCAP/Vector.

When the MOI value increased from 5 to 70, the transfection efficiency was enhanced from 2% to >90% 48 h after transfection (Fig. 3A and B). When the MOI value was 70, the SCAP/Vector and SCAP/BMP2 presented a high GFP expression proportion 4 days following gene transfection. However, the morphology of the transfected stem cells was slightly slimmer (Fig. 3C) as compared to SCAP. The *BMP2* gene expression was higher in SCAP/BMP2 (6.23±0.04) than in SCAP/Vector (1.00±0.12) (P<0.01) 4 days after transfection (Fig. 3D). The western blot analysis confirmed that BMP2 expression in SCAP/BMP2 was significantly increased compared with SCAP/Vector (Fig. 3E).

### Characterization of cell proliferation

**Figure 4:**
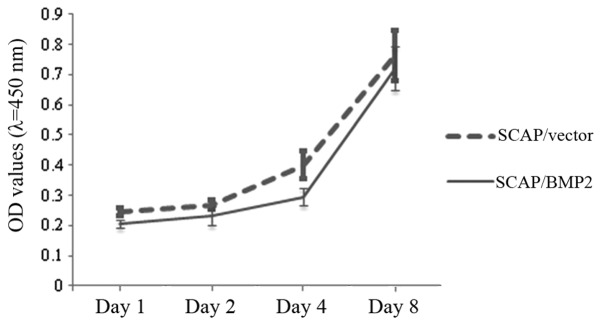
Cell proliferation status of stem cells from apical papilla (SCAP)/Vector and SCAP/BMP2 was analyzed by cell counting kit-8 (CCK-8) on the 1st, 2nd, 4th and 8th day following transfection. The cell proliferation status of SCAP/Vector was slightly more improved than SCAP/bone morphogenetic protein 2 (BMP2) at the four time-points.

No significant difference of cell proliferation was found between SCAP/Vector and SCAP/BMP2 (Fig. 4). The average optical density (OD) of the two groups of cells generally increased from 0.2 to 0.8 during the observation period.

### Odontogenic differentiation gene expression

**Figure 5:**
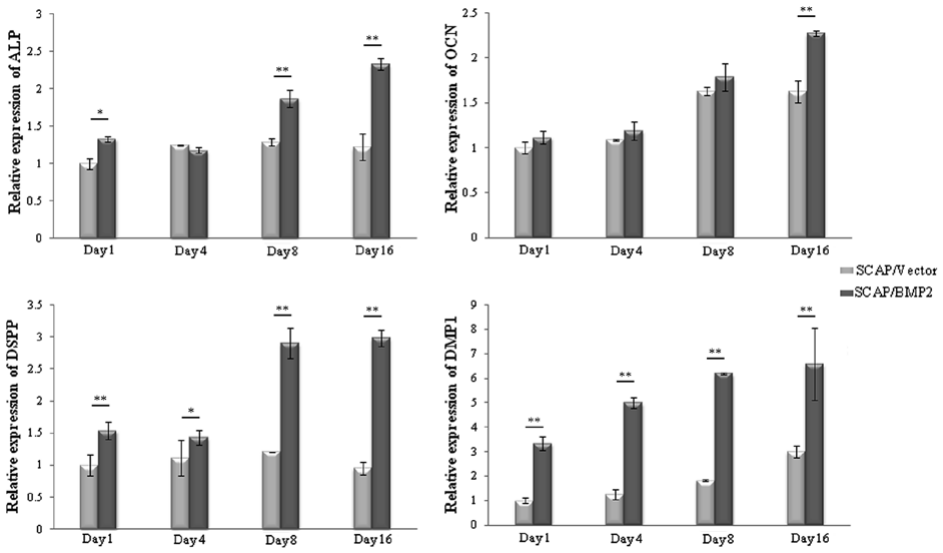
Odontogenic differentiation gene expression. Alkaline phosphatase (ALP), osteocalcin (OCN), dentin sialophosphoprotein (DSPP) and dentin matrix protein 1 (DMP1) gene relative expression of stem cells from apical papilla (SCAP)/Vector and SCAP/bone morphogenetic protein 2 (BMP2) were tested by quantitative polymerase chain reaction (qPCR) on the 1st, 4th, 8th and 16th day after lentiviral-mediated transfection. The relative expression of *ALP*, *OCN*, *DSPP* and *DMP1* genes was markedly enhanced in SCAP/BMP2 compared with SCAP/Vector. The statistical significance was marked as ^*^P<0.05 and ^**^P<0.01.

The relative peak expression of ALP in SCAP/Vector was on the 8th day after transfection, while for SCAP/BMP2 the expresion was on the 16th day. The ALP expression in SCAP/BMP2 was significantly enhanced on the 1st day (P<0.05), 8th day (P<0.01) and 16th day (P<0.01) compared with SCAP/Vector (Fig. 5). The relative peak expression of OCN in SCAP/Vector was on the 8th day, while in SCAP/BMP2 it was on the 16th day. The OCN expression in SCAP/BMP2 was significantly upregulated on the 16th day after gene transfection (P<0.01) compared with SCAP/Vector. The relative peak expression of DSPP in SCAP/Vector was on the 8th day, while in SCAP/BMP2 it was on the 16th day. The DSPP expression in SCAP/BMP2 was significantly upregulated on the 1st (P<0.01), 4th (P<0.05), 8th (P<0.01) and 16th (P<0.01) day compared with SCAP/Vector. The relative peak expression of DMP1 in SCAP/Vector and SCAP/BMP2 was on the 16th day. The DMP1 expression in SCAP/BMP2 was significantly upregulated at the four time points (P<0.01) compared with SCAP/Vector.

### ALP staining

**Figure 6:**
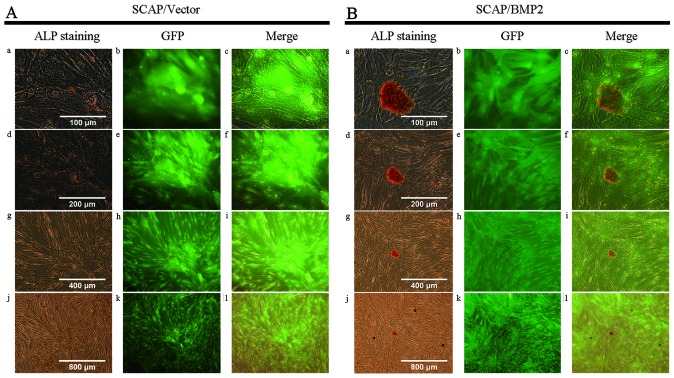
Alkaline phosphatase (ALP) staining. The ALP staining of stem cells from apical papilla (SCAP)/Vector and SCAP/bone morphogenetic protein 2 (BMP2) was performed on the 16th day after lentiviral-mediated transfection. A positive ALP expression was defined as golden staining granules and strips in the cells. (Ba–c) The ALP staining was observed in SCAP/Vector and SCAP/BMP2. Particularly, ALP staining was observed in the mineralized deposit that formed by SCAP/BMP2 at ×800 magnification. (Bd–f) The quantity of ALP staining in SCAP/BMP2 at (Ad–f) ×400 magnification was significantly more than that of SCAP/Vector. (Bg–l) More and larger mineralized deposits were detected in SCAP/BMP2 than (Ag–l) SCAP/Vector at ×100 and ×200 magnification. The GFP expression in the SCAP/Vector and SCAP/BMP2 was evident, suggesting that the cells maintained high transfection efficiency after 16 days culture *in vitro*.

A positive ALP expression, defined as golden staining granules and strips, was observed in both the SCAP/Vector and SCAP/BMP2 (Fig. 6). The quantity and brightness of ALP staining in SCAP/BMP2 was greater than that of SCAP/Vector. ALP expression was observed in intracellular as well as extracullar mineralized deposits.

### AR staining

**Figure 7:**
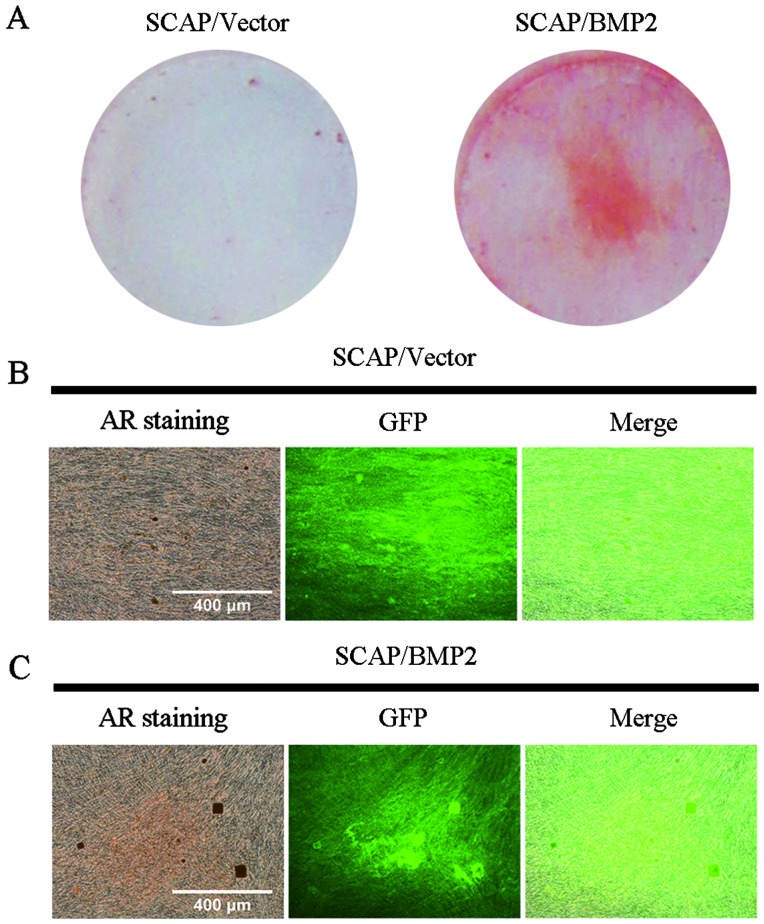
Alizarin red (AR) staining. The AR staining of stem cells from apical papilla (SCAP)/Vector and SCAP/bone morphogenetic protein 2 (BMP2) was performed on the 16th day after transfection. (A) AR staining in SCAP/BMP2 was significantly stronger than that in SCAP/Vector subsequent to visual inspection. (B and C) SCAP/BMP2 formed significantly more mineralization deposits than SCAP/Vector. The mineralization deposits had a strong green fluorescent protein (GFP) expression in SCAP/BMP2 and SCAP/Vector.

The SCAP/BMP2 demonstrated significantly stronger AR staining compared with SCAP/Vector on visual inspection (Fig. 7). More and larger mineralization deposits were observed in SCAP/BMP2 compared with SCAP/Vector under a microscope. For SCAP/BMP2, an intensive GFP expression was found in the mineralized deposits.

## Discussion

Dental papilla is commonly regarded as the source for tooth formation and ultimately converts to dentin and dental pulp tissue (1). SCAP, as a novel population of multipotential stem cells isolated from the dental papilla, have the capacity to differentiate into osteoblasts, odontoblast-like cells and adipocytes *in vitro* (1–7). Sonoyama *et al* induced the osteo/odontogenic differentiation of SCAP by using basic culture medium (α-MEM and 15% FBS) plus 10 nmol/l dexamethasone, 10 mmol/l β-glycerophosphate, 50 μg/ml ascorbate phosphate, and 10 nmol/l 1,25 dihydroxyvitamin D3 (1,5). SCAP formed mineralized deposits progressively after 4–8 weeks of induction. In the present study, the osteogenic differentiation of SCAP was induced using Human Mesenchymal Stem Cell Osteogenic Differentiation Medium which contains a similar formula to that described by Sonoyama *et al*. Mineralized deposits were formed following induction for 32 days. Sonoyama *et al* induced the adiogenic differentiation of SCAP by using the basic culture medium plus 0.5 mmol/l hydrocortisone, 60 mmol/l indomethacin, 10 mg/ml insulin and 0.5 mmol/l isobutylmethylxanthine (1). Results of those studies showed that the adipogenic differentiation capacity of SCAP was not as qualitative as bone marrow mesenchymal stem cells, and few lipid droplets were formed after 3 weeks. In the present study, lipid droplet formation of SCAP was induced with Human Mesenchymal Stem Cell Adipogenic Differentiation Medium for 8 days. The results of the present study confirmed that SCAP have the capacity for osteogenic and adipogenic differentiation.

SCAP were found to express MSC phenotypes, including STRO-1, CD146, CD24, CD105, CD73, CD90, CD29, CD44 and CD49 (4). STRO-1, a marker that is recognized as a trypsin insensitive epitope of perivascular cells, has been used to isolate MSC populations from dental pulps and apical papilla (4,13). STRO-1 selected DPSCs and SCAP showed an enhanced ability of osteo/odontogenic differentiation. Findings of previous studies have shown that the expression of STRO-1 among SCAP is usually >18%. Additionally, the STRO-1 expression of passage 1 SCAP is up to 20–30% (4,7). CD146 is one of the most employed key markers to characterize perivascular multipotent stem cells in connective tissues. The range of CD146 among SCAP is 47.3–84.8% (3). In the present study, the representative expression of STRO-1 of passage 2 SCAP was 21.8%, while the expression of CD146 was 60.3%. These results are consistent with those of previous studies. CD24 is considered a specific marker for SCAP, which is undetectable in other MSCs, including DPSCs (1). The CD24 expression of SCAP ranges from 3.2 to 15.3% (4), and its expression decreases with an upregulated expression of ALP during the odontogenic differentiation of SCAP (1). In the present study, the CD24 expression of passage 2 SCAP was 12.2%, which is consistent with that identified in previous studies. The FCM results confirmed that the phenotypes of the cells isolated from the apical papilla in this study are characteristic of SCAP.

Stem cells cultured with dexamethasone have been confirmed to form more mineralized deposits *in vitro* (20). Stem cells cultured with L-ascorbic acid-2-phosphate have also been reported to result in an upregulated cell proliferation and osteogenic differentiation (20–22). Culturing cells with mineralization stimuli may partly obscure the positive effects, which may due to *BMP2* gene transfection. Therefore, the cell-culturing media without external supplements of dexamethasone or any other mineralization stimuli were specifically selected for the present *in vitro* study.

In tooth development, BMP2 was found to express during the whole process of differentiation and maturation of odontoblasts (8,9). BMP2 is crucial in the regulation of odontoblast differentiation and dentin formation. *BMP2* gene transfection has been considered an effective strategy in the improvement of the odontogenic differentiation potential of DPSCs *in vitro* (13,14,16), leading to a significantly increased gene expression of *ALP*, *OCN*, *COL I*, *BSP*, *DSP* and *DMP1*. In the present study, the odontogenic differentiation mRNA expression, including ALP, OCN, DSPP and DMP1, was also significantly upregulated in SCAP/BMP2 compared with SCAP/Vector. Although in this study, BMP2 expression was only tested on the 4th day following gene transfection in, other studies have proven that lentivirus transfection can mediate a stable expression of target genes in cells (23–25).

ALP, which was detected during the process of cell mineralization, is considered an early marker of osteo/odontogenic differentiation of stem cells derived from dental tissues (2,3,11,13,26–30). Results of the present study have shown that SCAP/BMP2 had significantly upregulated ALP expressions on the 1st, 8th and 16th day compared with SCAP/Vector, which was similar to that in DPSCs/BMP2 (13). Additionally, ALP and AR staining demonstrated that SCAP/BMP2 formed more ALP granules and mineralized deposits compared with SCAP/Vector. OCN is a marker of the late stages of osteo/odontogenic differentiation, which regulates the mineral phase of bone and dentin (3). In a previous study it was found that DPSCs/BMP2 had a significantly upregulated expression of OCN on the 8th and 16th day after transfection (13), while in the present study SCAP/BMP2 showed a significantly upregulated expression of OCN on the 16th day. DSPP is a significant part of the dentin non-collagenous proteins, which play a key role in the dentin mineralization process (3,26). It is usually synthesized by terminally differentiated odontoblasts during the secretory phase, and is regarded as a late-stage marker of odontogenic differentiation. In the present study, qPCR showed that lentiviral-mediated *BMP2* gene transfection significantly enhanced the expression of DSPP gene at the four time-points, which was similar to a previous report (13). DMP1 is an acidic phosphorylated extracellular matrix protein (12,31). Although DMP1 is not exclusively expressed in odontogenic differentiation, it has been considered an important marker for odontoblasts (26,32). DMP1 has dual functions, as a transcription factor that targets the nucleus and as an extracellular matrix protein that initiates mineralization (29). In the present study, DMP1 expression in SCAP/BMP2 was significantly improved, which was also consistent with a previous report (13). These results provided evidence that the specific transfection of the *BMP2* gene enhanced the odontogenic differentiation of SCAP.

In summary, human dental papilla stem cells were successfully transfected by *BMP2* gene lentiviral plasmid. Under the circumstances without mineralization stimuli, SCAP/BMP2 expressed more odontogenic differentiation genes, revealed more ALP granules, and formed more mineralization deposits than SCAP/Vector. These results are consistent with our hypothesis. Transfection of a homologous *BMP2* gene may therefore be an effective strategy to improve the tissue engineering applications of SCAP.

## Figures and Tables

**Table I:** Primer sequences used in qPCR.

Gene	Sense	Antisense	Size (bp)
*BMP2*	CACTGTGCGCAGCTTCC	CCTCCGTGGGGATAGAACTT	107
*ALP*	CTATCCTGGCTCCGTGCTC	GCTGGCAGTGGTCAGATGTT	100
*OCN*	CTCACACTCCTCGCCCTATT	TTGGACACAAAGGCTGCAC	107
*DSPP*	GCCACTTTCAGTCTTCAAAGAGA	GCCCAAATGCAAAAATATGTAA	130
*DMP1*	AAAATTCTTTGTGAACTACGGAGG	GAGCACAGGATAATCCCCAA	94
*GAPDH*	AAGGTGAAGGTCGGAGTCAA	AATGAAGGGGTCATTGATGG	108

BMP2, bone morphogenetic protein 2; ALP, alkaline phosphatase; OCN, osteocalcin; DSPP, dentin sialophosphoprotein; DMP1, dentin matrix protein 1; GAPDH, glyceraldehyde-3-phosphate dehydrogenase.
